# Autoantibodies, Oxidative Stress, and Nutritional State in Anorexia Nervosa

**DOI:** 10.3390/antib14010001

**Published:** 2024-12-24

**Authors:** Andrea Amerio, Eleonora Martino, Antonella Strangio, Andrea Aguglia, Andrea Escelsior, Benedetta Conio, Samir Giuseppe Sukkar, Daniele Saverino

**Affiliations:** 1 Department of Neuroscience, Rehabilitation, Ophthalmology, Genetics, Maternal and Child Health (DINOGMI), Section of Psychiatry, University of Genoa, 16132 Genova, Italy; andrea.amerio@unige.it (A.A.);; 2 IRCCS Ospedale Policlinico San Martino, 16132 Genova, Italy; 3 Dietetics and Clinical Nutrition Unit, Genoa University, 16132 Genoa, Italy; 4 Department of Experimental Medicine (DiMeS), Section of Human Anatomy, University of Genoa, 16132 Genova, Italy

**Keywords:** anorexia nervosa, autoantigens, hypothalamus, oxidative stress, uric acid, total antioxidant capacity

## Abstract

**Background/Objectives**: Anorexia nervosa (AN) is a complex psychiatric disorder characterized by an extreme fear of gaining weight, leading to severe calorie restriction and weight loss. Beyond its psychiatric challenges, AN has significant physical consequences affecting multiple organ systems. Recent research has increasingly focused on the interplay between autoantibodies, oxidative stress, and nutritional state in this condition. **Methods**: Ninety-six subjects were evaluated: forty-eight with AN and forty-eight normal-weight control subjects. The serum levels of IgG reactive to hypothalamic antigens, uric acid, and total antioxidant capacity were evaluated by laboratory assays. **Results**: Anti-hypothalamic autoantibodies were found in AN patients. Furthermore, increased levels of oxidative stress were reported, as measured by decreased serum uric acid and total antioxidant capacity (TAC), and they reduced with the disease duration and the restoration of body mass index (BMI). Finally, a decrease in both autoantibodies and oxidative stress was observed as patients’ clinical condition improved, as measured by time since diagnosis and BMI recovery. **Conclusions**: The clinical improvement of AN patients seems to be associated with a decrease in the autoimmune response to hypothalamic cellular antigens and a reduction in oxidative stress. Dysregulation of the immune system and oxidative stress appear to be interconnected in various diseases, including autoimmune and psychiatric disorders. These findings, although preliminary, may offer potential avenues for the treatment of this challenging condition.

## 1. Introduction

Eating disorders (EDs) are debilitating mental health conditions characterized by abnormal eating patterns, weight control behaviors, and a distorted perception of body image [1]. They represent a significant public health concern due to their association with medical complications and psychiatric comorbidities [1,2]. In fact, EDs are among the deadliest mental illnesses worldwide, with anorexia nervosa (AN) having the highest mortality rate [2,3,4].

AN is characterized by a restrictive diet, an intense fear of gaining weight, and a distorted body image. It typically affects adolescents and young women and has a chronic course. Individuals with AN often engage in compulsive physical exercise to control their weight. The prevalence of anorexia nervosa varies, but it is generally estimated to be 0.3–1% in women and 0.1–0.3% in men. Nevertheless, the actual prevalence may be higher, as many cases go undiagnosed or unreported. The underlying causes of AN are complex and still not fully understood, making it difficult to develop effective therapeutic responses [5,6].

Inflammation has been implicated in the development of EDs, particularly AN [7,8,9]. Recent studies have shown altered immune-inflammatory states in both anorexic patients and animal models of AN. Increased levels of pro-inflammatory cytokines, such as interleukin-1 beta (IL-1β), IL-6, IL-15, and tumor necrosis factor-alpha (TNF-α), have been reported in individuals with AN. Conversely, levels of transforming growth factor-beta (TGF-β) are often lower [7,8,9].

A potential role of immune system dysfunction in the pathogenesis of AN has been suggested [5,9,10,11,12,13], supported by the presence of autoantibodies in individuals with AN [5,9,10,11,12,13]. However, the precise mechanisms through which these autoantibodies exert their effects remain unclear. Autoantibodies could potentially interact with specific ligands expressed by the hypothalamic cells, interfering with their normal functions. Alternatively, they may induce non-specific stimulation of target cells, leading to increased secretion of anorexigenic molecules. The production of autoantibodies directed against regulatory peptides and/or hypothalamic neurons could contribute to an appetite disorder characterized by reduced food intake. In individuals with AN, autoantibodies against several appetite-regulating peptides have been detected. However, the significance of this association is often difficult to interpret. While autoantibodies against key appetite-regulation hormones or neuropeptides have been found in healthy individuals, the presence of EDs may be related to the quantity and affinity of these autoantibodies [5,9,10,11,12,13]. Therefore, the development of AN could be triggered by the infiltration of these high-affinity autoantibodies into brain centers.

Oxidative stress may also contribute to the progression and severity of AN, being associated with several pathophysiological processes, including inflammation [14,15]. A recent study found increased oxidative stress in the plasma, urine, and saliva of AN patients [16], while a meta-analysis conducted by Solmi and colleagues has shown increased levels of redox mediators in patients with AN, which can be improved through weight restoration [17].

Although the role played by uric acid remains unclear, as does its association with psychiatric disorders [18], it can be envisaged that uric acid in AN may play a more active role than previously thought [15,16,17,19]. Given the limited research on uricemia in AN and the complex nature of uric acid homeostasis, a crucial initial step is to investigate serum uric acid levels in large cross-sectional and longitudinal studies of patients with AN. Given the strong evidence linking uric acid to key features and mechanisms of AN, including weight regulation, oxidative stress, immune dysfunction, and mood disturbances, further research is needed to explore its potential as a therapeutic target for this disorder.

The aim of this study is to confirm the presence of autoantibodies addressed to hypothalamic cells in patients with AN compared to healthy controls during different phases of the illness (i.e., time from the diagnosis, weight restoration). Moreover, while highly speculative, we propose several promising avenues for future research delving into the complex relationship between oxidative stress and AN.

## 2. Materials and Methods

### 2.1. Patient Recruitment and Enrollment

Forty-eight patients with AN were enrolled voluntarily in this study between October 2019 and July 2024, and written informed consent was signed. AN diagnosis was made according to the criteria of the *Diagnostic and Statistical Manual of Mental Disorders, Fifth Edition (DSM-5)* [1]. Participants were provided with an in-depth explanation of the study objectives and procedures with the opportunity to ask questions about the goals of this study. Blood samples were collected in the morning (between 7.30 and 9.30 am, before breakfast), at least six hours after the last meal. Serum samples were stored frozen until analysis. Freezing and thawing were avoided. Finally, a follow-up of 12 patients was performed at diagnosis and 6 months later, during the recovery (BMI ≥ 18.5). This study was approved by the Ethical Committee of the Istituto di Ricovero e Cura a Carattere Scientifico (IRCCS) Ospedale Policlinico San Martino (CER 82/13 Emend. 028, 2 March 2017), and all participants provided written informed consent. The study was conducted in accordance with the Declaration of Helsinki II [20]. A total of 48 healthy donors (HDs) were selected for the study based on an interview that ruled out individuals with a history of eating disorders or autoimmune diseases.

### 2.2. ELISA Protocol for Anti-Hypothalamus Autoantibodies Detection

Serum samples from patients with AN and healthy controls were analyzed for the presence of IgG antibodies specific to hypothalamic antigens using a homemade direct ELISA method [13]. In brief, 96-well plates were coated with bovine hypothalamic lysate and blocked with bovine serum albumin to prevent non-specific binding. Diluted serum samples were added to the plates and incubated overnight. Anti-human IgG-conjugated horseradish peroxidase (HRP) was then added, followed by a colorimetric substrate. A standard curve for human IgG was established using purified human IgG. This standard curve was used to quantify the concentration of anti-hypothalamus autoantibodies in the serum samples. The optical density readings of the serum samples were converted into IgG concentrations in µg/mL. The intra-assay coefficient of variation (CV) was 5%, and the inter-assay CV was 9.3%. The deviation between triplicates was less than 10% for all reported values. The formula used for a cut-off value calculation was as follows: cut-off index value (CO) = mean OD negative samples + 2 standard deviation. Accordingly, a positive result was mean OD/CO ≥ 2.0, whereas a negative one was mean OD/CO < 2.

Sensibility and specificity were calculated following the results in Table 1. Sensibility was 0.88 and specificity was 1.

### 2.3. ELISA Test for Uric Acid and Total Antioxidant Capacity Evaluation

Uric acid was assessed via enzyme-linked immunosorbent assays (ELISA, EMELCA Bioscience, Kapucinessenstraat 30; B-2000 Antwerp, Belgium), following the manufacturers’ instructions. The assay range was 617.3–50,000 µg/dL and the sensitivity 243.1 µg/dL; the intra-assay CV was <10% and the inter-assay CV was <12%.

In addition, antioxidant capacity was measured by spectrophotometric assays using the General Total Antioxidant Capacity Assay Kit (TAC, EMELCA Bioscience; Kapucinessenstraat 30; B-2000 Antwerp, Belgium) in serum samples. The total antioxidant capacity (TAC) assay measures total antioxidant capacity in which Fe^3+^-TPTZ is reduced by antioxidants to Fe^2+^-TPTZ. The enzyme-catalyzed reaction product Fe^2+^-TPTZ can be measured at a colorimetric readout at 593 nm. The assay range was 0.05–5 nmol/mL.

### 2.4. Statistical Analysis

To assess the normality of data distribution, the D’Agostino–Pearson normality test was employed [21]. This test evaluates Skewness and Kurtosis to determine how closely the distribution resembles a Gaussian distribution in terms of asymmetry and shape. A single *p*-value is calculated based on the combined deviations of these values from the expected Gaussian distribution.

The Mann–Whitney U-test was used to compare levels of autoreactive IgG, uric acid, and TAC oxidative markers. The Wilcoxon test was utilized to analyze differences in the concentrations of these parameters based on disease duration (<3 years vs. ≥3 years). Spearman’s correlation analysis was used to assess the correlation between different parameter levels in AN patients. Finally, we performed an independent samples T-test to compare the mean age and BMI of the two independent groups. A *p*-value of less than 0.05 was considered statistically significant. All analyses were performed using GraphPad Prism 6.0 (GraphPad Software Inc., La Jolla, CA, USA) software.

## 3. Results

### 3.1. Characteristics of the Study Population

Of the 48 participants, 40 had the restricting subtype of AN, while 8 had the purging phenotype (vomiting or use of laxatives). All participants were female (except for two), aged between 18 and 62 with an average age of 20.8 ± 9.1. The body mass index (BMI) of the participants was 15.2 ± 1.9. A control group (CG) of 44 females and 4 males, matched for age, was also included in the study. These individuals were not affected by eating disorders or autoimmune diseases. Their average age was 23.1 ± 4.61, and their BMI was 22.5 ± 0.7. Table 2 shows the characteristics stratified according to BMI.

### 3.2. Anti-Hypothalamus Autoantibodies

**Figure 1:**
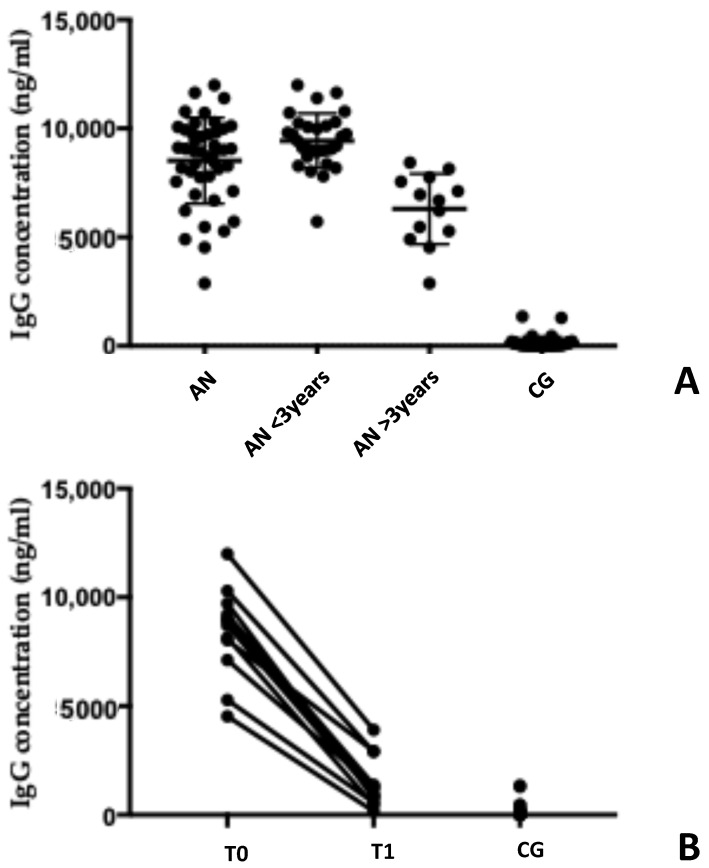
Serum anti-hypothalamus autoantibodies in patients with AN are remarkably increased compared to healthy subjects. Serum anti-hypothalamus autoantibodies (Ig G) are significantly elevated in AN patients compared to healthy individuals. Panel (**A**) illustrates the difference in autoantibody levels between AN patients and healthy controls. Furthermore, a decrease in autoantibody levels was observed over time since diagnosis. Panel (**B**) shows the results of a follow-up study in 10 patients, comparing autoantibody levels at diagnosis (T0) and 6 months later (T1). A significant decrease in autoantibody levels was observed. AN: anorexia nervosa patients; CG: control group.

There was a significant over-representation of serum anti-hypothalamus autoantibodies in patients with AN compared to healthy subjects: mean IgG 8522 ng/mL ± 1978 (range 2883–11,988 ng/mL) for patients with AN vs. 144.2 μg/mL ± 283.1 (range 30–1350 ng/mL) for CG (*p* < 0.001) (Figure 1 and Table 2). Examining the graphical distribution of Ig G autoantibody levels in the serum of patients with AN (Figure 1A), a distinction into two different subgroups is evident. An analysis of the characteristics of patients within these two clusters revealed that the significant differences were the duration of the observed AN: less or more than 3 years, corresponding to a difference in the BMI (mean 14.6 ± 1.9 and 18.3 ± 1.0, respectively). This suggests that the duration of AN and the restoration of the BMI (and the correct nutritional state) may influence the serum levels of Ig G autoantibodies. Indeed, Ig G autoantibody levels decreased over time, approaching those of the healthy subjects (Figure 1B). Specifically, patients with AN for less than 3 years (and BMI < 17) had higher Ig G autoantibody levels (9.44 ng/mL ± 1.26) compared to those with AN for more than 3 years (and BMI > 17) (6303 ng/mL ± 1616) (*p* < 0.001). The control group had a baseline level of 144.2 μg/mL ± 283.1. While the differences between the Ig G autoantibody levels in patients with AN for more than 3 years and the control group were still statistically significant (*p* = 0.001), as shown in Figure 1A, the overall trend was towards a decrease. Despite the limited number of male participants, no clear differences were observed between males and females.

In a follow-up study of 12 patients diagnosed with anorexia nervosa (AN), we monitored their levels of autoantibodies over a six-month period. As illustrated in Figure 1B, there was a significant decrease in autoantibody levels during this timeframe. The mean autoantibody levels dropped from 8.42 ng/mL ± 2.05 to 1.45 ng/mL ± 1.45 (*p* = 0.001), indicating a highly statistically significant reduction. As shown in Figure 1B, the comparison of IgG autoantibody concentrations between the AN group after recovery and the healthy subjects was statistically different (*p* < 0.001). However, the mean concentration of autoantibodies in recovered patients approached that of controls (1450 ± 1.45 ng/mL vs. 144.2 ± 283.1 ng/mL, respectively).

### 3.3. Oxidative Status Evaluation: Uric Acid and Total Oxidative Content

**Figure 2:**
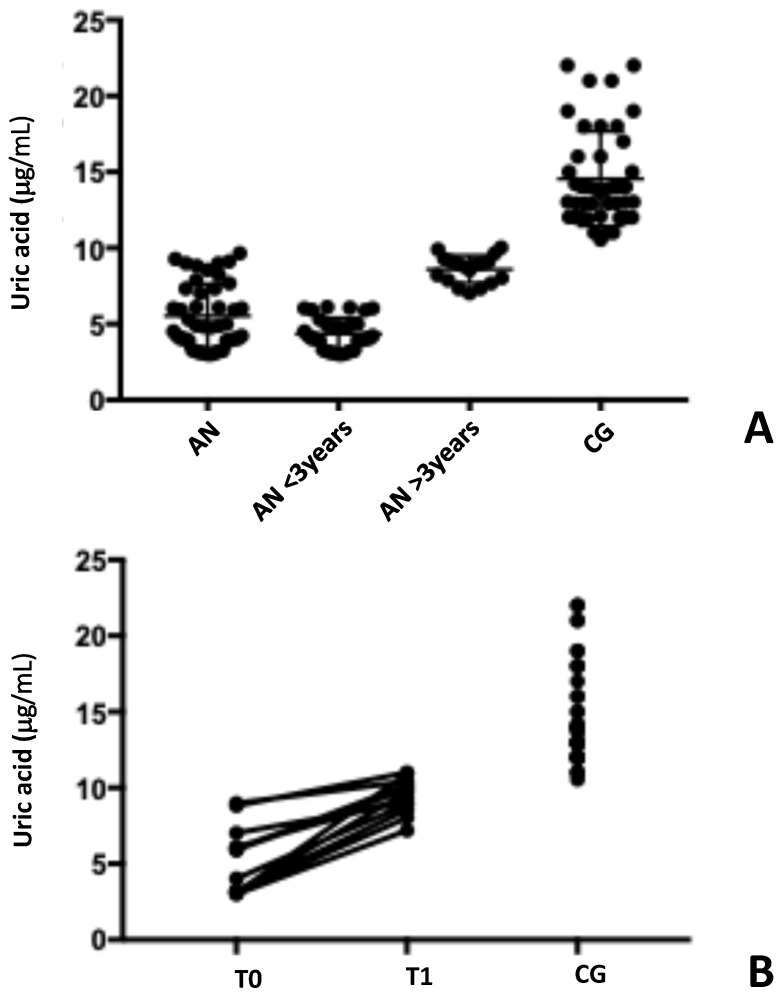
Serum uric acid levels in patients with AN are significantly decreased compared to healthy subjects. Serum uric acid levels are significantly lower in AN patients compared to healthy individuals. Panel (**A**) illustrates the difference in uric acid levels between AN patients and healthy controls. Furthermore, an increase in uric acid levels was observed over time since diagnosis. Panel (**B**) shows the results of a follow-up study in 10 patients, comparing uric acid levels at diagnosis (T0) and 6 months later (T1). A significant increase in levels was observed. AN: anorexia nervosa patients; CG: control group.

Serum uric acid values are shown in Table 2 and Figure 2.

A significant reduction in serum uric acid levels between patients with AN and the healthy subjects (5.5 μg/mL ± 2.1 vs. 14.6 μg/mL ± 3.1, *p* < 0.001) was found. Additionally, the time elapsed since diagnosis was reported to have a significant impact on uric acid levels, suggesting a gradual decrease in oxidative stress as time from diagnosis and nutritional status improved (4.35 μg/mL ± 1.05 vs. 8.60 μg/mL ± 0.91, *p* < 0.001) (Figure 2A).

Furthermore, a significant decline in uric acid values was observed in the six-month follow-up (5.27 μg/mL, ± 0.60 vs. 9.49 μg/mL, ± 0.31, *p* = 0.001) (Figure 2B).

**Figure 3:**
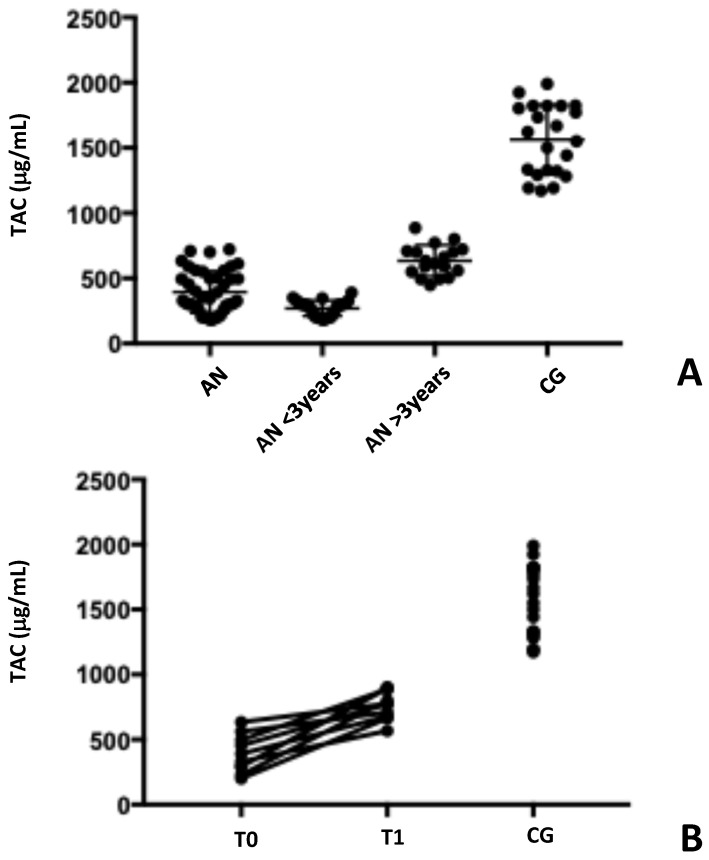
Serum TAC levels in patients with AN are significantly decreased compared to healthy subjects. Serum TAC levels are significantly lower in AN patients compared to healthy individuals. Panel (**A**) illustrates the difference in TAC levels between AN patients and healthy controls. Furthermore, an increase in TAC levels was observed over time since diagnosis. Panel (**B**) shows the results of a follow-up study in 10 patients, comparing TAC levels at diagnosis (T0) and 6 months later (T1). A significant increase in levels was observed. AN: anorexia nervosa patients; CG: control group; TAC: total antioxidant capacity.

Total antioxidant capacity (TAC) reflects the body’s overall defense against oxidative stress, a condition caused by harmful molecules known as free radicals. The TAC values for the study participants are shown in Table 2 and Figure 3.

When comparing AN patients to healthy subjects, we found a significant difference in their TAC levels (399.4 μmol/L ± 164.0 vs. 1575 μmol/L ± 273.2, respectively, *p* < 0.001). This suggests that AN patients may have a weaker ability to protect themselves from oxidative stress.

Based on previous results, AN patients were clustered in two different groups. The differences in the TAC levels measured (285.7 μmol/L ± 95.57 vs. 635.3 μmol/L ± 119.2, *p* < 0.001) reinforce the hypothesized distinction between the two groups (Figure 3A). This indicates that the duration of the disorder, linked to the recovery of nutritional state (evaluated as BMI increasing) may affect how well the body can handle oxidative stress. As patients with AN recover and improve their nutrition, their TAC levels tend to decrease, suggesting a reduction in oxidative stress.

Additionally, in the six-month follow-up, a significant reduction in their TAC levels was found (406.0 μmol/L ± 161.0 vs. 793.4 μmol/L, ±137.8, *p* < 0.001) (Figure 3B). This result supports the hypothesis that improving nutrition and overall health can help to reduce oxidative stress in patients with AN.

### 3.4. Investigation of a Possible Correlation Between Autoantibody Levels and Oxidative Stress

**Figure 4:**
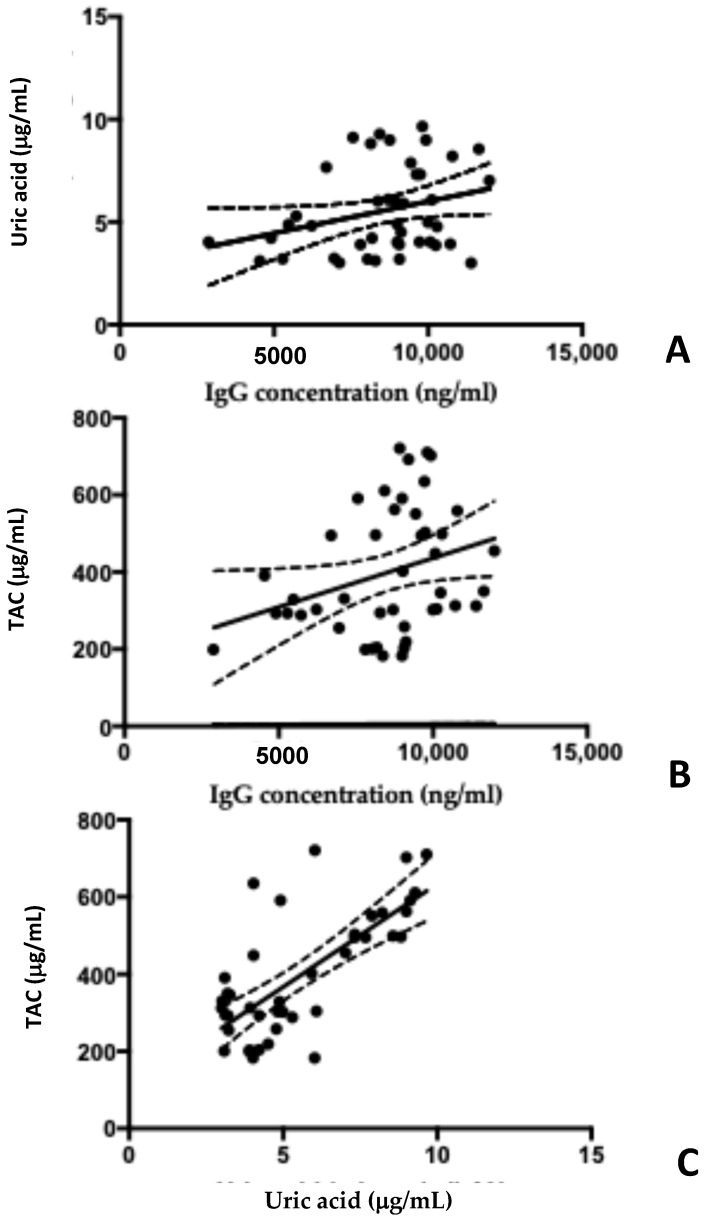
Correlation analysis between autoantibodies and oxidative stress. Panel (**A**) suggests a possible weak correlation between autoantibody (IgG) levels and uric acid concentration. Panel (**B**) also shows a positive correlation between autoantibody levels and TAC. Panel (**C**) demonstrates a significant positive correlation between uric acid and TAC, confirming the presence of oxidative stress in AN patients. TAC: total antioxidant capacity.

A preliminary observation of Figure 4A,B suggests a positive correlation between autoantibody concentrations and uric acid and/or TAC, leading to an incomplete conclusion. In fact, there is no statistically significant correlation between the levels of autoantibodies measured in the serum of AN patients and uric acid (R squared 0.07, *p* = 0.050) or TAC levels (R squared 0.09, *p* = 0.043). It is likely that a link exists between oxidative stress and the dysregulation of the immune system, but it is not possible to demonstrate this unequivocally. Obviously, uric acid levels and TAC levels are correlated, supporting the hypothesis that the two markers measure the oxidative stress characterizing these patients (R squared 0.56, *p* < 0.001).

## 4. Discussion

Patients with AN often have disordered eating patterns that can persist even after receiving outpatient treatment. This highlights the need for improved nutritional counselling and weight rehabilitation strategies [22], confirmed by studies that showed significant micronutrient deficiencies in AN patients [23].

In this study, the primary focus of patient recovery was on nutritional rehabilitation and weight restoration, which are essential goals in the early treatment of AN [24]. These recovery parameters were likely closely linked to changes in antioxidant activity. The average time to recovery (BMI ≥ 18.5) was variable: 4.1 ± 2.4 months, which is consistent with typical recovery times in similar programs, where patients often regain near-normal weight within six months [25,26,27]. For this reason, autoantibodies and antioxidant state were evaluated after six-month follow-up.

The growing evidence suggests a link between immune system disruptors and the development of autoimmune diseases and EDs, confirming a higher prevalence of autoimmune diseases [5,10,11], and suggesting shared immunological pathways that may connect these two types of conditions. Autoimmunity could potentially play a role in triggering or suppressing EDs, at least in a specific subgroup of patients (whose molecular characteristics are currently unknown). Recent studies have reported cases of AN associated with juvenile systemic lupus erythematosus [28], Hashimoto’s thyroiditis [29], celiac disease [30,31], and inflammatory bowel disease [32,33]. Thus, a reciprocal relationship, with individuals affected by one condition being more likely to develop the other, could exist. Common factors contributing to this link include inflammation, immune system irregularities, and the presence of autoantibodies. Although existing studies have not fully mapped out the specific immunological pathways connecting AN and autoimmune diseases, understanding their interplay is crucial. Identifying these shared mechanisms could potentially reclassify AN as a psycho–neuro–endocrine–immune disorder. While the immune component might be complex to pinpoint, focusing on both neuropsychiatric and immunological aspects could be key to improving treatment outcomes.

Recent findings highlight a strong relationship between the changes caused by oxidative stress and the immune response [34]. Additionally, chronic inflammation and disruptions in immune balance can lead to more severe oxidative stress and damage to cells and tissues [34].

When the body’s ability to produce antioxidants cannot keep up with the production of harmful substances called free radicals, it can lead to various health problems [35,36]. A poor diet can significantly increase the production of free radicals [37,38]. Previous research has shown that TAC is a reliable indicator of the overall antioxidant status in the blood and reflects both dietary antioxidant intake and the body’s natural antioxidant defenses [35,36,37,38,39,40].

Primarily associated with gout, a common inflammatory arthritis, uric acid can also be an uncommon complication of anorexia nervosa [41]. Although uric acid may be involved in the underlying mechanisms and psychological aspects of anorexia nervosa, research in this field has largely centered on its role as a metabolic waste product. AN is an uncommon risk factor for conditions like tophi and gout [41]. These conditions may often be overlooked in AN patients due to the typical association of gout with factors such as a high-purine diet, obesity, and older age [42]. Conversely, malnutrition, vegetarian diets, and low-purine diets are linked to low uric acid levels (hypouricemia) [43]. The conventional understanding of the relationship between AN and uric acid has been unidirectional, with dietary factors and physiological responses to eating disorders affecting serum uric acid levels. However, recent research suggests a more complex, bidirectional relationship, where personality traits, behavioral patterns, and serum uric acid levels may interact and influence each other [44,45].

Our study investigated the relationship between nutritional status (assessed by changes in BMI, time since diagnosis, and clinical evaluation), autoantibody production, and oxidative stress in AN patients. Previous research suggests that immune system disruptors may play a role in both autoimmune diseases and eating disorders, potentially through shared immunological pathways [5,8,9,10,11,12,13,14]. Furthermore, patients with AN who underwent a re-nutrition program and achieved a normal BMI experienced improvements in TAC and uric acid serum concentration. This suggests that TAC and uric acid levels may be useful indicators of nutritional status in these patients. A possible explanation is that an adequate diet and improved nutritional status can lead to a healthier cellular redox state. In fact, malnutrition has been linked to the increased production of reactive oxygen species.

Overall, re-nutrition appears to restore the antioxidant defense system, enabling it to better cope with the increased free radical production observed in AN. Furthermore, the time elapsed since diagnosis and the improvement of malnutrition conditions are correlated with a decrease in circulating autoantibodies against hypothalamic receptors. Although it is difficult to definitively prove a direct causal relationship between the presence of autoantibodies and increased oxidative stress, it is certain that the inflammatory state characteristic of AN, along with the presence of circulating autoantibodies, induces an oxidative stress that becomes self-perpetuating over time. A return to a correct dietary state could potentially interrupt this vicious cycle and restore balance in patients affected by AN.

It is noteworthy that anti-hypothalamus autoantibodies have been linked to various diseases, primarily those affecting the endocrine system, but not exclusively [46]. The involvement of these autoantibodies in the pathogenesis of AN appears to be increasingly relevant. In fact, the hypothalamus, a critical brain area governing various bodily functions, including hormone production and release, can be affected by autoimmune attacks, potentially leading to health complications.

One limitation of this study is the relatively small sample size of follow-up patients (n = 12) participating. However, this is one of the largest studies to date on patients with AN (n = 48) that has examined oxidative stress biomarkers and autoantibodies in these patients. In addition, this study focused on female patients, enrolling only two male patients. Future research should investigate these parameters in male individuals to determine if sex influences the results. It is worth noting that the majority of AN patients are female, accounting for 90% of cases [47,48], but that male patients are increasing.

## 5. Conclusions

In conclusion, the relationship between autoantibodies and oxidative stress is often bidirectional [49,50]. Autoantibodies can contribute to oxidative stress by damaging tissues and triggering inflammatory responses. Conversely, oxidative stress can promote the production of autoantibodies by altering immune cell function and increasing inflammation.

The interconnectedness of anorexia, autoimmunity, and oxidative stress underscores the urgent need for a comprehensive therapeutic approach. Further research is necessary to fully understand whether the observed relationship is a cause or a marginal aspect in the pathogenesis of AN. By addressing the root causes of these conditions, we can significantly improve the lives of those affected.

A deeper understanding of the role of immune dysregulation and oxidative stress could pave the way for novel therapeutic approaches. The administration of immunosuppressive drugs could be considered in the acute phase of AN, mitigating the negative effects of autoantibodies on hypothalamic cells. Concurrently, supplementation with antioxidants (e.g., vitamin C, vitamin E, zinc, manganese, selenium, copper, or riboflavin) could alleviate oxidative stress. It is important to note that these interventions should not replace conventional treatment (where psychotherapy plays a key role), but rather serve as complementary therapies.

Finally, our findings suggest a potential role for immune dysregulation and oxidative stress in the pathophysiology of this disease. Additional prospective studies are required to assess the efficacy of therapeutic interventions targeting immune dysregulation and oxidative stress in improving prognosis and quality of life.

## Figures and Tables

**Table 1:** Evaluation of the sensibility and sensitivity of the homemade ELISA test.

	Positive	Negative	Total
Anorexia nervosa	48 (a)	0 (b)	48
Control group	6 (c)	42 (d)	48
Total	54	42	56

Sensibility = a/a + c; Specificity = d/d + b.

**Table 2:** Comparison between patients with AN and healthy control group in terms of sociodemographic and oxidative markers.

	Anorexia Nervosa (*N* = 48)	Control Group (*N* = 48)	*p*-Value
Gender (male/female)	2/46	4/44	-
Age (years)	20.8 ± 9.1	23.1 ± 4.61	0.45
Body mass index (kg/m^2^)	15.2 ± 1.9	22.3 ± 0.7	<0.001
Serum markers			
IgG autoantibody to hypothalamic cells (ng/mL)	8522 ± 1978	144.2 ± 283.1	<0.001
Uric acid (μg/mL)	5.5 ± 2.1	14.6 ± 3.1	<0.001
TAC (μmol/L)	399.4 ± 164.0	1575 ± 273.2	<0.001

## Data Availability

Data that support the findings of this study and materials are available from the corresponding author upon request.
